# Smoking Experimentation among Elementary School Students in China: Influences from Peers, Families, and the School Environment

**DOI:** 10.1371/journal.pone.0073048

**Published:** 2013-08-22

**Authors:** Cheng Huang, Jeffrey Koplan, Shaohua Yu, Changwei Li, Chaoran Guo, Jing Liu, Hui Li, Michelle Kegler, Pam Redmon, Michael Eriksen

**Affiliations:** 1 Department of Global Health, School of Public Health and Health Services, George Washington University, Washington, District of Columbia, United States of America; 2 Global Health Institute, Emory University, Atlanta, Georgia, United States of America; 3 Department of Criminology and Criminal Justice, Georgia State University, Atlanta, Georgia, United States of America; 4 Department of Epidemiology, Tulane University, New Orleans, Louisiana, United States of America; 5 Department of Health Policy and Management, Rollins School of Public Health, Emory University, Atlanta, Georgia, United States of America; 6 New York State Division of Criminal Justice Services, Office of Justice Research and Performance, Albany, New York, United States of America; 7 Chinese Centre for Disease Control and Prevention at Ningbo, Ningbo, Zhejiang, China; 8 Department of Behavioral Sciences & Health Education, Rollins School of Public Health, Emory University, Atlanta, Georgia, United States of America; 9 Institute of Public Health, Georgia State University, Atlanta, Georgia, United States of America; The University of Auckland, New Zealand

## Abstract

The aim of this study was to investigate experimentation with smoking among primary school students in China. Data were acquired from a recent survey of 4,073 students in grades 4 to 6 (ages 9–12) in 11 primary schools of Ningbo City. The questions were adapted from the Global Youth Tobacco Survey (GYTS). Results suggest that although the Chinese Ministry of Education (MOE) encourages smoke-free schools, experimentation with cigarettes remains a serious problem among primary school students in China. Peers, family members, and the school environment play important roles in influencing smoking experimentation among students. Having a friend who smoked, seeing a family member smoke, and observing a teacher smoking on campus predicted a higher risk of experimentation with smoking; the exposure to anti-tobacco materials at school predicted a lower risk of experimentation with smoking. The evidence suggests that public health practitioners and policymakers should seek to ensure the implementation of smoke-free policies and that intervention should target young people, families, and communities to curb the commencement of smoking among children and adolescents in China.

## Introduction

Youth smoking remains a serious public health problem despite the recent decline in some countries, including the US, where substantial resources have been devoted to curb the pandemic [1–3]. The Global Youth Tobacco Survey (GYTS), a school-based survey of students aged 13–15 conducted in 131 countries between 1999 and 2005, showed that 8.9% of students currently smoked; and one in five nonsmoking students reported that they were likely to smoke by the following year [4]. Several national surveys suggested that adolescent smoking increased rapidly during the past few decades in China [5–7]. For example, smoking prevalence among men aged 15–19 increased from about 18% in 1996 to 21% in 2002 despite a decrease in smoking prevalence in the general Chinese population by 1.8% during the same period [6]. A study conducted in 2007 on passive smoking and its risk factors among 25,600 adolescents in 32 regions of China showed that 6.3% currently smoked, 23.1% had experimented with smoking, and 57.5% had been exposed to secondhand smoke [8]. Of particular concern is that the early initiation of smoking increases the likelihood of smoking dependence in adults [9–11] and causes more negative health outcomes. For example, starting to smoke before the age of 15 doubles one’s risk of developing lung cancer compared to starting to smoke at the age of 20 [12,13].

Multiple theoretical models have been developed to explain the initiation of adolescent smoking [14,15]. Among the most influential were Leventhal and Cleary’s four-stage smoking onset construct—preparation, initiation, becoming, and maintenance [16]—and Flay’s five-stage model—preparatory stage, initial trying, experimentation, regular use, and nicotine dependence [17]. During the preparation stage, children or adolescents are exposed to influences and opportunities to smoke, gain knowledge, and form beliefs and expectations about smoking and its effects. During initiation or initial trying, children become involved in smoking for the first time, smoking only a few cigarettes. Gradually, such children or adolescents indulge in smoking as a regular but still infrequent habit. Once they reach the maintenance or nicotine dependence stage, they smoke daily or almost daily, and their attempts to quit trigger withdrawal symptoms [18]. Similar to the development of other deviant behaviors through “social learning,” adolescent smoking behavior is acquired, in part through observing the behavior of role models in the social environment [19]. Experimentation may be directly influenced by the attitudes and smoking behaviors of their parents, siblings, teachers, and peers [20–24]. The school provides a crucial setting where social learning may take place [24]. In a school where smoking restrictions are absent and teachers smoke, students may perceive smoking as socially acceptable and therefore may be more likely to smoke [24].

Although evidence suggests that the majority of smokers start using tobacco between the ages of 11 and 13 [25], very little research has focused on elementary school students in China. Exceptions include an earlier investigation conducted in 1988 in Beijing [26]. In past decades the tobacco industry has increasingly advertised their products to young people through multiple channels. Many primary schools have been funded by tobacco companies as part of China’s Hope Project, a charity that was initiated to support schools in poor rural areas [27]. Such sponsorship by the tobacco industry may support a positive image of tobacco, promote the sale of tobacco products, and jeopardize the implementation of school-based interventions [28]. The Chinese government, however, signed and ratified the WHO Framework Convention on Tobacco Control (WHO FCTC) in 2005 and has subsequently initiated some programs to curb the smoking pandemic and reduce exposure to secondhand smoke. In particular, in 2010 the Ministry of Health (MOH) and the Ministry of Education (MOE) jointly released guidelines for schools to establish smoke-free campuses [29]; however, the extent to which the MOH and MOE have implemented or enforced these policies remains unclear.

In this study, we used data from a recent survey of elementary school students in Ningbo, China, and investigated smoking experimentation among youths aged 9–12, a younger group than those investigated in most previous studies, including the GYTS. We examined how smoking experimentation among elementary school students was associated with tobacco-related knowledge and attitudes, smoking behaviors of their parents, peers, and teachers as well as smoking policies and programs of the schools they attended.

## Methods

### Study population

Ningbo is an eastern coastal city in China with a total population of 2.21 million. In 2009 it was one of the 17 cities selected to participate in the Emory Global Health Institute–Tobacco-Free Cities (TFC) program, funded by the Bill & Melinda Gates Foundation and aimed at changing the social norms regarding tobacco use in China [30]. Each city participating in TFC implemented interventions on target populations, including pregnant women, government employees, healthcare providers, and students, with a strong emphasis on creating smoke-free environments. TFC administrators in Ningbo targeted students in elementary and junior high schools. At the outset of the project, a survey was conducted to obtain tobacco-related benchmark information on elementary school students in Ningbo’s six districts, including Haishu, Jiangdong, Jiangbei, Beilun, Zhenhai, and Yinzhou, from which 11 primary schools were chosen using stratified cluster sampling, with six in urban areas, three in suburban areas, and two in rural areas. All students in Grades 4 through 6 in these 11 schools were included in the study. Based on the GYTS [31], the survey was adapted to reflect the context of China. Efforts were made to ensure that the survey participants understood the questions; for example, *Pinyin*, the phonetic version of Chinese using the Roman alphabet, was added to clarify difficult Chinese characters. The survey was approved by the Institutional Review Board of the Ningbo Centers of Disease Control (CDC). Enumerators from the CDC, who received training before the administration of the survey, strictly followed procedures. The survey was anonymous, and passive consent was obtained from the participants and their parents. Teachers were not present while students completed the survey, and each of the returned questionnaires was checked for completeness by the enumerators [30].

### Measurement

#### Dependent variable

We measured smoking experimentation with a question directly adopted from the GYTS: “Have you ever tried or experimented with cigarette smoking, even one or two puffs?” Those who answered “yes” were defined as having experimented with smoking.

#### Independent variables

Predictors of smoking experimentation were adopted from the GYTS and pertained to the following topics: smoking-related knowledge and attitudes, familial/home environment factors, peer factors, and school-related factors that potentially affect smoking behaviors among adolescents [7,8,11–13]. Specifically, smoking-related knowledge and attitudes were measured with the following questions: Do you think smoking tobacco is harmful to your health? Do you think the smoke from other people’s tobacco smoking is harmful to you? and Do you agree or disagree with the following: I think I might enjoy smoking a cigarette? The peer-related questions included the following: Do you think boys who smoke cigarettes have more or fewer friends? Do you think girls who smoke cigarettes have more or fewer friends? and Do any of your closest friends smoke tobacco? These six questions were also adopted directly from GYTS.

We measured familial/home environment factors with the following question: Do your parents (or stepparents/guardians who stay at your home) smoke? and added several ad hoc questions including, Do you think it’s polite to distribute cigarettes to guests visiting your home? Do you think your parents will mind you smoking when you are older? and During the past 30 days, have you seen any antismoking media messages on television? Previous research has suggested that cigarette gifting and sharing in China, which is culturally acceptable, may increase the risk of adolescent smoking [32]. In addition, antismoking mass media campaigns appear to be effective in preventing the initiation of smoking in early adolescence [33] and deserve further investigation given that Chinese children are now spending up to more than three hours per day watching television, primarily at home [34].

Smoking in the school environment was measured by the following question: Did you see any of your teachers smoke inside the school building or outside on the school property last week? This question resembles others on the GYTS (e.g., During school hours, how often do you see teachers smoking in the school building? During school hours, how often do you see teachers smoking outdoors on school premises?). Additional questions related to tobacco and the school environment included the following: Did your school distribute any tobacco control educational materials to you last semester? In the past semester, did anyone teach you skills on refusing smoking at school? and Do you see anti-smoking information on campus very often?

### Analysis

The original sample consisted of 4,315 students in Ningbo, ranging in age from 8 to 17. We restricted our analysis to students aged 9–12 years, comprising 4,073 individuals (94.4% of original sample): 2,286 boys and 1,787 girls. We further excluded cases with missing values for any variables included in the analysis.

Logistic regression analyses were used to estimate the crude associations between experimentation with smoking and each category of risk factors in separate models (Model 1–4), controlling for sex, grade, and location of the school. We tested sex differences in the associations between a predictor and the outcome by including a “sex * predictor” interaction in each model. In Model 5, we also assessed the independent effect of each category of risk factors, controlling for other categories. We reported odds ratios (ORs) and their 95% confidence intervals (CI). The standard error was adjusted for clustering within a class because students in the same class were more likely to be exposed to similar antitobacco education and materials. All the analyses were conducted with Statistical Analysis Software (SAS), version 9.0.

## Results

The descriptive analysis (Table 1) suggests that the risk of experimentation with smoking was significantly different in boys and girls (p<0.001, not shown in the table). With respect to knowledge about the harmfulness of tobacco, 96.0% of boys and 96.8% of girls reported smoking was harmful, but only 80.4% of boys and 81.5% of girls thought secondhand smoke was harmful. Approximately 29.5% of boys and 25.7% girls thought that distributing cigarettes to guests was polite. Only 10.9% of boys and 7.6% of girls thought that their parents would not mind whether they smoked when they were older. The majority of students (68.8% of boys and 68.5% of girls) reported that some family member had smoked in front of them. Regarding the tobacco control practices at school, 30.3% of boys and 25.9% of girls had seen teachers smoke in the previous week, and more than 80% of students often saw antismoking information on campus. About 55% of students reported received tobacco control educational materials during the previous semester. Table 1presents additional descriptive information related to these variables by gender and smoking status; the characteristics differ significantly by smoking status, especially among boys.

**Table 1:** Characteristics of the sampled students in elementary schools at Ningbo, by sex and smoking status.

Variables	Boys (%)			Girls (%)		
	Smoked	Never	Diff	Smoked	Never	Diff
	(n=445)	(n=1,841)	P	(n=134)	(n=1,653)	P
School area						
*Urban*	52.4	63.1	<.0001	59.0	65.6	0.1219
*Suburban/ Rural*	47.6	36.9		41.0	34.4	
Grade						
*4^th^*	36.6	35.8		37.3	34.8	
*5^th^*	38.2	33.4	0.0403	41.8	35.9	0.1088
*6^th^*	25.2	30.9		20.9	29.3	
Age						
*Mean (SD)*	10.6 (1.0)	10.5 (1.0)	0.5344	10.4 (1.1)	10.4 (1.0)	0.5721
Do you think smoking tobacco is harmful to your health?						
*No*	7.0	3.2		6.0	3.0	
*Yes*	93.0	96.7	0.0003	94.0	97.0	0.0642
*Missing*	0	0.1		0	0	
Do you think the smoke from other people’s tobacco smoking is harmful to you?						
*No*	23.2	18.6		27.6	17.7	
*Yes*	76.4	81.3	0.0265	71.6	82.3	0.0037
*Missing*	0.5	0.1		0.8	0.1	
Do you agree or disagree with the following: “I think I might enjoy smoking a cigarette.”						
*Agree*	7.2	3.3		3.0	1.8	
*Disagree*	92.8	96.6	0.0002	96.3	98.3	0.3019
*Missing*	0	0.1		0.8	0	
Do you think it is polite to distribute cigarettes to guests visiting your home?						
*No*	60.7	72.6		61.2	75.1	
*Yes*	39.3	27.2	<.0001	38.1	24.7	0.0005
*Missing*	0	0.2		0.8	0.2	
Do you think your parents will mind you smoking when you are older?						
*No*	13.9	10.2		11.2	7.3	
*Yes*	61.6	75.1	<.0001	70.2	81.5	0.0058
*Do not know*	24.5	14.7		18.7	11.2	
Do your parents (or stepparents/guardians who stay at your home) smoke?						
*They do not smoke*	7.4	14.8		10.5	14.5	
*Smoke, but not in front of the respondent*	5.2	7.6		4.5	9.7	
*Smoke in front of the respondent*	76.6	66.9	<.0001	79.9	67.6	0.0232
*Missing*	10.8	10.7		5.2	8.2	
During the past 30 days, have you seen any anti-smoking media messages on television?						
*No*	11.0	9.8	0.4365	9.7	9.2	0.8457
*Yes*	89.0	90.2		90.3	90.8	
Do you think boys who smoke cigarettes have more or less friends?						
*More*	14.6	6.3		10.5	5.8	
*Less*	62.5	75.7	<.0001	70.2	77.1	0.0627
*No difference*	22.7	17.8		19.4	17.0	
*Missing*	0.2	0.2		0	0.1	
Do you think girls who smoke cigarettes have more or less friends?						
*More*	7.6	2.2		2.2	1.5	
*Less*	79.1	87.7	<.0001	86.6	90.7	0.4197
*No difference*	13.3	10.0		10.5	7.8	
*Missing*	0	0.1		0.8	0	
Do any of your closest friends smoke tobacco?						
*No*	50.6	84.8		69.4	91.8	
*Yes*	44.5	15.1	<.0001	24.6	8.2	<.0001
*Missing*	4.9	0.1		6.0	0	
Did you see any of your teachers smoke inside the school building or outside on school property last week?						
*No*	55.1	73.0		56.7	75.4	
*Yes*	44.7	26.9	<.0001	43.3	24.5	<.0001
*Missing*	0.2	0.2		0	0.1	
Did your school distribute any tobacco control educational materials to you last semester?						
*No*	57.3	42.3	<.0001	61.9	43.8	<.0001
*Yes*	42.7	57.7		38.1	56.2	
In the past semester, did anyone teach you skills on refusing smoking at school?						
*No*	75.5	64.7	<.0001	71.6	69.3	0.5759
*Yes*	24.5	35.4		28.4	30.7	
Do you see anti-smoking information on campus very often?						
*No*	21.1	19.5		22.4	17.5	
*Yes*	78.4	80.3	0.3682	77.6	82.4	0.1606
*Missing*	0.5	0.2		0	0.1	

Table 2 presents the results of the logistic regression models. As shown in Model 1, elementary school students in suburban and rural areas had a 50% higher risk of experimentation with smoking than their counterparts in urban areas (OR=1.50; 95% CI: 1.03-2.18). The risk of experimentation with smoking among girls was significantly lower than that among boys (OR=0.34; 95% CI: 0.27-0.43); moreover, lack of awareness that smoking was harmful (OR=1.96; 95% CI: 1.28-3.00) and indifference to teenage smoking (OR=1.40; 95% CI: 1.15-1.71) predicted a higher risk of experimentation with smoking.

**Table 2:** Estimated odds ratio (95% confidence interval) of experimentation of smoking among primary school students.

Variables	Model 1-Knowledge & attitude	Model 2- Family Effect	Model 3- Peer Effect	Model 4- School Environment	Model 5- Full Model
School area (Urban)					
Suburban/Rural	1.50 (1.03-2.18)*	1.39 (0.97-1.98)	1.13 (0.81-1.57)	1.30 (0.93-1.81)	0.90 (0.66-1.22)
Grade (4^th^)					
5^th^	1.19 (0.76-1.86)	1.11 (0.69-1.78)	1.15 (0.76-1.76)	1.12 (0.74-1.71)	1.12 (0.74-1.70)
6^th^	0.85 (0.55-1.30)	0.85 (0.55-1.30)	0.75 (0.51-1.09)	0.84 (0.53-1.32)	0.74 (0.49-1.13)
Gender (Boy)					
Girl	0.34 (0.27-0.43)*	0.35 (0.28-0.44)*	0.41 (0.32-0.51)*	0.34 (0.27-0.42)*	0.41 (0.32-0.52)*
Do you think smoking tobacco is harmful to your health? (Yes)					
No	1.96 (1.28-3.00)*				1.52 (1.02-2.28)*
Do you think the smoke from other people’s tobacco smoking is harmful to you? (Yes)					
No	1.21 (0.92-1.60)				1.13 (0.87-1.47)
Do you agree or disagree with the following: “I think I might enjoy smoking a cigarette.” (Disagree)					
Agree	1.40 (1.15-1.71)*				1.22 (0.97-1.54)
Do you think it is polite to distribute cigarettes to guests visiting your home? (No)					
Yes		1.78 (1.35-2.36)*			1.52 (1.16-1.98)*
Do you think your parents will mind you smoking when you are older? (Yes)					
No		1.47 (1.05-2.05)*			1.18 (0.84-1.65)
Do not know		1.67 (1.16-2.39)*			1.44 (1.02-2.04)*
Do your parents (or stepparents/guardians who stay at your home) smoke? (They do not smoke)					
Smoke, but not in front of the respondent		1.02 (0.62-1.69)			0.93 (0.57-1.54)
Smoke in front of the respondent		1.86 (1.35-2.55)*			1.64 (1.21-2.21)*
During the past 30 days, have you seen any anti-smoking media messages on television? (Yes)					
No		1.01 (0.87-1.18)			0.97 (0.83-1.13)
Do you think boys who smoke cigarettes have more or less friends?(less)					
More			1.93 (1.37-2.72)*		1.44 (1.02-2.03)*
No difference			1.16 (0.86-1.55)		0.92 (0.67-1.27)
Do you think girls who smoke cigarettes have more or less friends?(less)					
More			2.74 (1.63-4.59)*		2.48 (1.47-4.18)*
No difference			1.21 (0.78-1.88)		1.26 (0.81-1.96)
Do any of your closest friends smoke tobacco? (No)					
Yes			4.47 (3.37-5.94)*		3.91 (2.88-5.30)*
Did you see any of your teachers smoke inside the school building or outside on school property last week? (No)					
Yes				1.87 (1.49-2.35)*	1.52 (1.22-1.90)*
Did your school distribute any tobacco control educational materials to you last semester? (Yes)					
No				1.55 (1.16-2.09)*	1.46 (1.07-1.99)*
In the past semester, did anyone teach you skills on refusing smoking at school? (Yes)					
No				0.99 (0.83-1.17)	0.99 (0.82-1.19)
Do you see anti-smoking information on campus very often? (Yes)					
No				0.91 (0.69-1.20)	0.90 (0.67-1.20)
Model Fitness (-2 Log L)					
*Intercept only*	2878.9	2878.9	2878.9	2878.9	2878.9
*Intercept and covariates*	2716.3	2660.9	2528.6	2737.3	2448.2

As results from Model 2 suggest, the perception that it was polite to give cigarettes to guests (OR=1.78; 95% CI: 1.35-2.36) and having family members who had smoked in front of them (OR=1.86; 95% CI: 1.35-2.55) predicted a higher risk of smoking experimentation. In addition, the students who thought their parents would not mind whether they smoked when they were older (OR=1.47; 95% CI: 1.05-2.05) and the students who were not sure about their parents’ attitudes regarding their future smoking (OR=1.67; 95% CI: 1.16-2.39) were more likely to have experimented with smoking than those who thought that their parents would mind.

In Model 3 peer-related influences on experimentation with smoking were examined. All three risk factors, including the belief that boys who smoke have more friends (OR: 1.93; 95% CI: 1.37-2.72), the belief that girls who smoke have more friends (OR: 2.74; 95% CI: 1.63-4.59), and having close friends who smoked (OR=4.47; 95% CI: 3.37-5.94) predicted higher risks of experimentation with smoking.

In Model 4 the effect of school-based tobacco policies and programs were examined. Students who saw a teacher smoking on campus were more likely to have experimented with smoking (OR=1.87; 95% CI: 1.49-2.35). Students who reported that school personnel had not distributed antitobacco materials during the previous semester were more likely to have experimented with smoking (OR=1.55; 95% CI: 1.16-2.09) when compared with students who reported receiving the materials; however, neither frequent exposure to antismoking information on campus nor receiving education on refusal skills at school predicted the likelihood of experimentation with smoking.

Model 5 shows the associations of experimentation with smoking and all factors simultaneously. According to this model, the region (suburban/rural vs. urban) and the students’ attitude toward smoking no longer predicted the risk of smoking experimentation. Controlling for other factors attenuated the associations between the risk of experimenting with smoking and family-related effects, peer-related effects, and school effects; but most of these associations remained significant at the 0.05 level.

## Discussion

The rate of smoking experimentation for the sampled elementary school students in Ningbo was substantial, with an average rate of 19.5% among boys and 7.5% among girls. The significantly higher rate of experimentation with smoking among boys reflects gender differences in smokers in the adult Chinese population. According to the 2010 Global Adult Tobacco Survey (GATS), 52.9% of Chinese men in contrast to only 2.4% of women were current smokers [35]. Similar gender differences in smoking behaviors were found among other Asian students [36]. For example, a study reported that prevalence of smoking was higher among male students (22.3%) than among females (5.5%) in Malaysia [36]. Such a low smoking prevalence among females was likely related to cultural norms or stigma associated with female smoking [37]. Girls have also been found more likely to be “hidden smokers” in Asian culture [38]. This rate of experimenting with smoking is higher than that from a previous study conducted in 2006 in six Chinese cities, including Beijing, Shanghai, Tianjin, Shenzhen, Puyang, and Changsha, which showed that the rate of experimental smoking among similar-grade students was 10.0% among boys and 4.7% among girls [39]. The differing results in the two studies may simply reflect regional differences; however, evidence from many other countries including Asian region suggests that the rate of experimenting with smoking among elementary school students is rising [40–43]. A recent study in Taiwan showed that 34.9% of third- and fourth-grade students from mountain schools reported that they had tried cigarette smoking [44].

Prior research suggests that the initiation of smoking among adolescents may be related to parental and peer relations [11,45–48]. For example, adolescents who identified their parents as upset and thus responding negatively to their child smoking are less likely to smoke [49]. This is consistent with our findings that students who think their parents will not mind whether they smoked when they were older are more likely to have experimented with smoking. Our study also confirmed a positive association between experimenting with smoking and smoking behaviors of peers among very young students in Taiwan and South Korea [44,50]. Specifically, youth behaviors are heavily shaped by social interaction with peers; in fact, the utility of a given activity depends on the actions of their peers [51,52]. A friend who smokes may provide a young person with access to cigarettes or exert pressure on that individual to initiate smoking [44,50,53]. Although a youth may select friends who smoke because smoking peers are more accepting of others who smoke (selection effect), studies have provided evidence in support of the peer effect (i.e., having friends who smoke causally increases the risk of smoking initiation) instead of the selection effect [54,55]. Previous studies conducted in the US have shown that peer education programs that trained older students to become positive role models for elementary students were more effective compared to didactic programs led by researchers and teachers [56,57], and such studies may warrant further evaluation in the context of China.

Ample evidence suggests that the school environment plays an important role in smoking behaviors among students [57,58]. According to a national survey on knowledge, attitudes, and behaviors of teachers, 50.2% of male teachers in China smoke. The majority of these teachers (81.4%) smoke on campus, and 48.1% of them smoke in front of students [59]. Our results suggested that the smoking behaviors of teachers as role models significantly increase the risk of smoking experimentation among students. In 2010, the Chinese MOH and MOE jointly issued a policy in which smoking would be completely prohibited on elementary, junior high, and high school campuses [29]; however, the present study, based on data from the latest survey in Ningbo, suggests that this policy has not been effectively translated into practice.

In summary, findings from this study suggest that regardless of the recent regulations released by the Chinese MOE and MOH aimed at creating smoke-free campuses in China, early exposure to smoking is likely to remain a serious public health problem among elementary school students; in addition, the “smoke-free” goal is unlikely to be achieved in the absence of strict implementation of such regulations. Specific guidelines are expected from the Ministries or other Chinese authorities regarding tobacco control measures, the training of personnel, and the monitoring and evaluation of implementation and cessation services offered to faculty, staff, and students who smoke [58,60]. In addition, our findings, along with lessons from numerous studies in other countries, suggest that the health-knowledge deficit and other factors, including smoking behaviors of parents and peers as well as positive attitudes towards smoking, are crucial to the initiation of smoking [1,61]. Interventions aimed at curbing the initiation of youth smoking may therefore require a more comprehensive approach that includes tobacco-free schools, smoke-free homes, and smoke-free public places as well as training young people to resist negative influences from their peers, families, and communities [1,61]. In particular, programs that identify youth with a propensity to use tobacco and prevent them from initiating smoking may be promising and warrant further evaluation [57].

This study has several limitations. First, the smoking behaviors were self-reported, and students may have been reluctant to disclose their experiences with smoking as suggested by previous studies comparing self-reports and biochemical measures [62]. Second, peers, family, and society as factors in this study are loosely defined groups of variables, which should not be interpreted equivalently as some “latent” factors generated from factor analysis or similar techniques. Third, despite having carefully consulted the GYTS design, the Ningbo study was designed primarily to gather benchmark information for the subsequent implementation of tobacco prevention programs at the sampled schools; and the questionnaire was therefore heavily modified to serve this purpose, considerably limiting the feasibility of comparing our findings with those from GYTS-based studies.
